# Supplementary material for: Integral gene drives for population replacement
Source: Biol Open. 2018 Nov 29;8(1):bio037762. doi: 10.1242/bio.037762 (PMC6361204; doi:10.1242/bio.037762)
Supplement: Supplementary information [file biolopen-8-037762-s1.pdf]

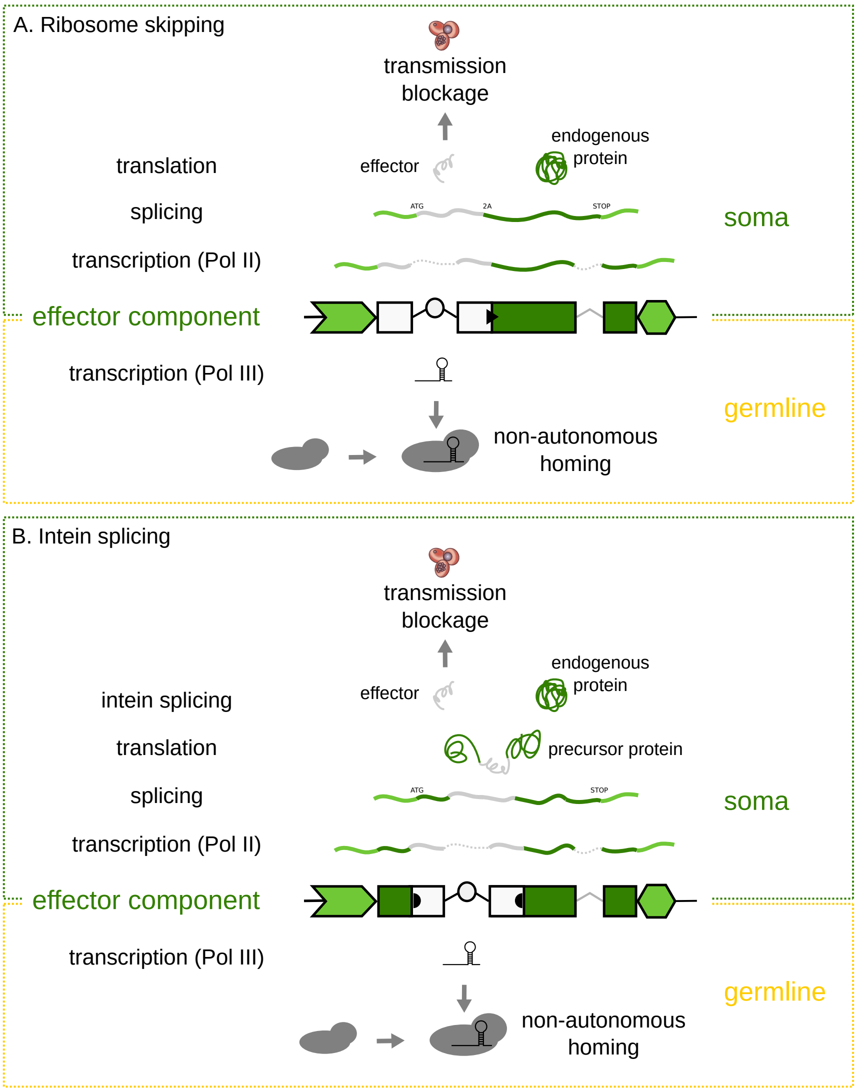

Figure S1. Schematic showing the expression of the endogenous and introduced genetic elements of an effector component allele at a hijacked gene locus.
Expression of the gRNA occurs in the germline (yellow) whereas expression of the hijacked gene occurs in an infection-relevant somatic tissue (green). While expressing a straight fusion of endogenous protein (green) and the effector protein (grey) is possible the generation of separate polypeptides by using either (A) the 2A ribosome-skipping signal (black triangle) or (B) the use of intein sequences (black semicircle) are possible avenues to avoid interfering with the function of the hijacked gene.

Biology Open • Supplementary information

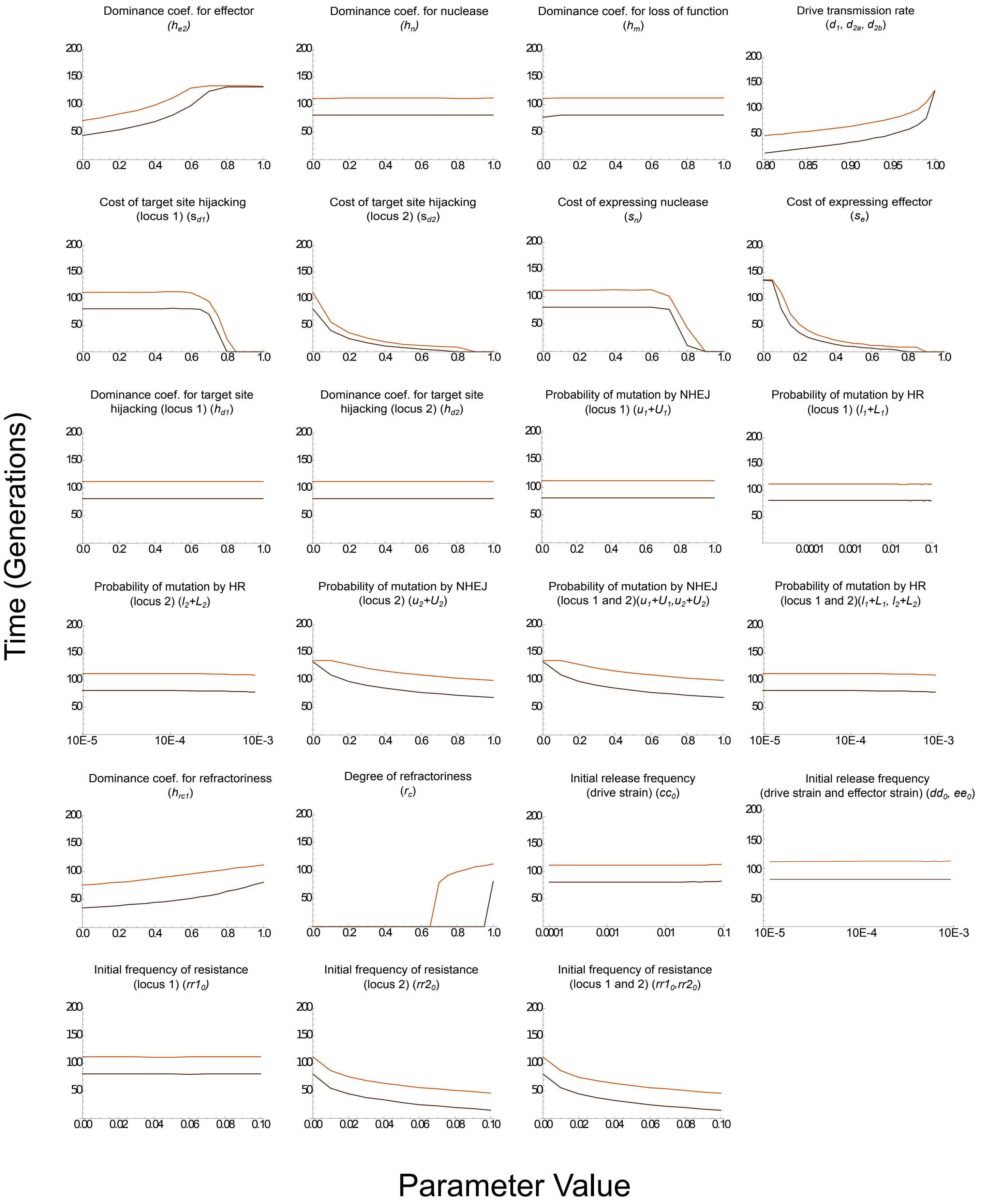

Figure S2. Duration of 67% and 95% protection (top and bottom lines in each graph) as each of the underlying parameters is varied, holding all others at their baseline values.

Biology Open • Supplementary information

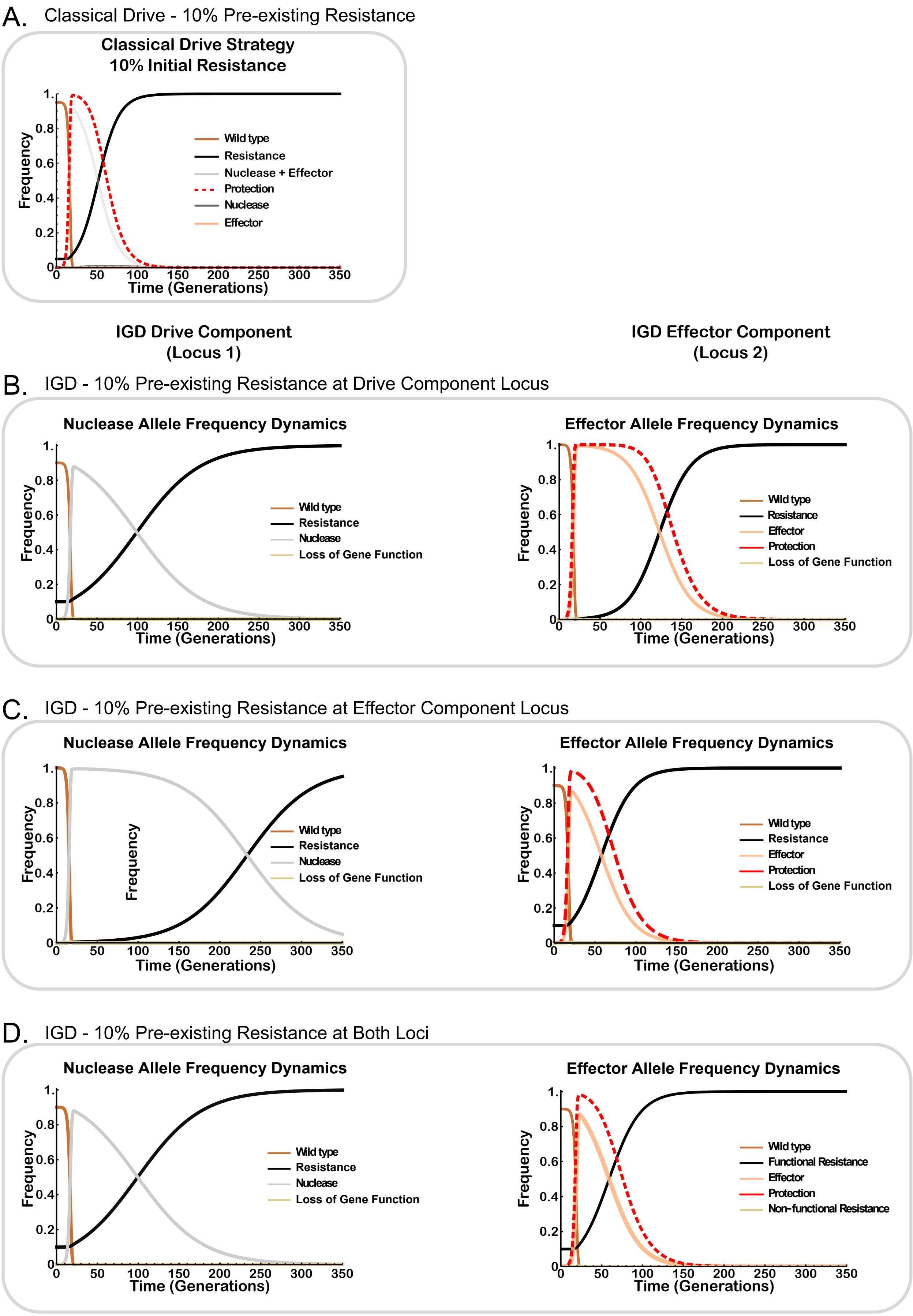

Figure S3. Overview of allele frequency dynamics when releasing both classical drive (A) and IGD strains into field populations with 10% pre-existing resistance.
For IGD, resistance is modelled at the drive component locus (B), the effector component locus (C), and at both loci (D).

Biology Open • Supplementary information
